# A synthesis of evidence on inhibitory control and auditory hallucinations based on the Research Domain Criteria (RDoC) framework

**DOI:** 10.3389/fnhum.2014.00180

**Published:** 2014-03-26

**Authors:** Johanna C. Badcock, Kenneth Hugdahl

**Affiliations:** ^1^Centre for Clinical Research in Neuropsychiatry, School of Psychiatry and Clinical Neurosciences, University of Western AustraliaCrawley, WA, Australia; ^2^Clinical Research Centre, North Metropolitan Health Service-Mental HealthPerth, WA, Australia; ^3^Division of Psychiatry, Department of Biological and Medical Psychology, NORMENT Centre of Excellence (RCN # 223273), Haukeland University Hospital, University of BergenBergen, Norway

**Keywords:** hallucinations, hearing voices, Research Domain Criteria project, inhibitory control, cognition

## Abstract

The National Institute of Mental Health initiative called the Research Domain Criteria (RDoC) project aims to provide a new approach to understanding mental illness grounded in the fundamental domains of human behavior and psychological functioning. To this end the RDoC framework encourages researchers and clinicians to think outside the [diagnostic] box, by studying symptoms, behaviors or biomarkers that cut across traditional mental illness categories. In this article we examine and discuss how the RDoC framework can improve our understanding of psychopathology by zeroing in on hallucinations- now widely recognized as a symptom that occurs in a range of clinical and non-clinical groups. We focus on a single domain of functioning—namely cognitive [inhibitory] control—and assimilate key findings structured around the basic RDoC “units of analysis,” which span the range from observable behavior to molecular genetics. Our synthesis and critique of the literature provides a deeper understanding of the mechanisms involved in the emergence of auditory hallucinations, linked to the individual dynamics of inhibitory development before and after puberty; favors separate developmental trajectories for clinical and non-clinical hallucinations; yields new insights into co-occurring emotional and behavioral problems; and suggests some novel avenues for treatment.

## Introduction

The National Institute of Mental Health (NIMH) Research Domain Criteria (RDoC) project aims to provide the foundations for a new approach to the classification and treatment of mental illness (Insel et al., 2010; Cuthbert and Insel, 2013). Specifically, the RDoC initiative was designed to provide “*new ways of classifying mental disorders based on dimensions of observable behavior and neurobiological measures*” (National Institute of Mental Health, 2013, p. 9). As an inherently dimensional system it naturally places psychiatric disorders on a continuum with normal experience, increasing the likelihood that they will be more understandable (Bentall, 1996).

The RDoC framework is easily visualized as a matrix of columns and rows^1^. The rows correspond to basic functional dimensions of human behavior (termed *Constructs*; see Table 1), grouped—at least at the moment—into five higher level *Domains:* Cognitive Systems, Systems for Social Processes, Negative and Positive Valence Systems, and Arousal/Regulatory Systems. The columns, on the other hand, reflect different levels of description or *Units of Analysis*, ranging from genetic, molecular and cellular levels, through neural circuitry and physiology on to observable behavior and self-report. Noticeably absent from this structure are developmental and environmental perspectives relevant to mental disorder, though the intent is the matrix “will enhance the study of both areas by promoting a systematic focus on their relationship to specific circuits and functions” (National Institute of Mental Health, 2013). In short, the RDoC project provides a scaffold to rapidly organize and integrate currently available evidence (Cole, 2013), and to think beyond traditional diagnostic boundaries by studying symptoms, behaviors or biomarkers that cut across many disorders. The time is ripe, therefore, for clinicians and researchers to take on the challenge and engage in a “constructive dialog” to move things forward (Insel et al., 2010, p. 750).

**Table 1:** **Higher order domains and associated constructs in the RDoC framework**.

**Cognitive systems**	**Arousal/modulatory systems**	**Positive valence systems**	**Negative valence systems**	**Systems for social processes**
Perception	Arousal	Approach motivation	Acute threat (fear)	Affiliation and attachment
Attention	Biological rhythms	Initial responsiveness to reward	Potential threat (anxiety)	Social communication
Working memory	Sleep/wake	Sustained responsiveness to reward	Sustained threat	Perception and understanding of self
Declarative memory		Reward learning	Loss	Perception and understanding of others
Language		Habit	Frustrative non-reward	
Cognitive control				

Hallucinations provide a really clear example of a symptom without borders, and are intimately linked with how we interact with others (Longden, 2010; Badcock and Chhabra, 2013). Whilst habitually identified as a sign of madness and schizophrenia, at least in contemporary western society (McCarthy-Jones, 2013), they are neither disorder nor disease specific. For example, auditory hallucinations (AH)—most often in the form of hearing “voices”^2^—occur in schizoaffective and bipolar disorder (Shinn et al., 2012); borderline personality disorder (Slotema et al., 2012; Schroeder et al., 2013); post-traumatic stress disorder (Jessop et al., 2008); dissociative identity disorder (Dorahy et al., 2009) and disorders of anxiety and depression (Varghese et al., 2011; Wigman et al., 2012). Hallucinations are also more common than is often realized in healthy individuals in the general community, including children, adolescents, adults and the elderly with no diagnosis of mental illness (Kelleher et al., 2012a,b; Laurens et al., 2012; de Leede-Smith and Barkus, 2013). Thus, AH provide a natural opportunity to test the advantages and limitations of the RDoC approach.

A quick search of the literature shows there has been a marked rise in the number of studies on the etiology of AH in the last decade (Blom and Sommer, 2012; Jardri et al., 2012; Badcock et al., 2014). Unitary process accounts of AH have rapidly been displaced in favor of multiple deficit models involving a combination of perceptual, cognitive, and socio-emotional processes (Badcock, 2010; Waters et al., 2012; Stephane, 2013) which can now be mapped to fill the rows (i.e., *Constructs*) of the RDoC matrix. Similarly, the evidence base on AH includes a wide variety of analytic techniques (genetic, pharmacological, neural circuitry, neurophysiology, behavior, and phenomenology) with which we can begin to populate the columns (i.e., *Units of Analysis*) of the matrix. The purpose of this paper is to illustrate this approach by assimilating recent evidence concerning one single construct associated with AH—namely Inhibitory Control (from the Cognitive Systems Domain). This choice is not meant to imply that this construct is solely or even primarily responsible for AH; rather it was chosen because this cognitive function has been implicated in generating socially flexible behavior and a key RDOC construct linked to AH (Ford et al., in press), and because it may be regarded as a gate-keeper that allows AH to persist once they are elicited, and not suppressed or inhibited. Thus, a selective focus on inhibitory control allowed us to integrate literature on both normal and abnormal functioning to gain a deeper understanding of the mechanisms involved in(auditory) hallucinations, the divergent trajectories to adult mental health outcomes (Kaymaz et al., 2012; Fisher et al., 2013) and the frequent co-occurrence of other emotional and behavioral problems. Finally, both strengths and gaps in the matrix are highlighted to serve as a guide for future research. Before continuing, however, it should be noted that a similar analysis could be done for the other domains of the RDoC matrix—including other constructs from the cognitive systems domain (e.g., perception and attention), which are critically involved in AH and show individual variation from normal to pathological behavior (see Hugdahl et al., 2009a,b) as well as domains which have been relatively understudied in relation to AH (e.g., Systems for Social Processes; see Ford et al., in press).

## Cognitive systems domain: construct cognitive control

The RDoC working group identified six constructs within the Cognitive Systems Domain: Cognitive Control, Perception, Attention, Declarative and Working Memory, and Language (see Table 1). Whilst each of these constructs is believed to be relevant to AH, the first is particularly salient from the perspective of voice hearers themselves since they typically do not *feel* they have direct, voluntary control over their perceptions (David, 2004), which can influence their take up of effective treatments (Freeman et al., 2013). Cognitive control can be thought of as the ability to exert top-down control over task relevant processes and/or coordinate thoughts and actions to achieve a particular goal (Miller and Cohen, 2001). It relies on a superordinate neural network, including prefrontal and parietal cortices that interact with subcortical areas, components of which may be differentially activated depending on the current task demands (Niendam et al., 2012).

### Subcomponent—inhibitory control

A fundamental aspect of cognitive control is the ability to inhibit or suppress irrelevant information or actions, so that a task-appropriate response can be made. Inhibition can take many forms and may best be described as a “family” of processes which vary in terms of what is being inhibited (e.g., thoughts or behavior) and how inhibition occurs (e.g., triggered by an external signal or driven by environmental context), each assayed by different types of task. In addition, there are large individual and age-related differences in inhibitory ability. Alternatively, defined in terms of stimuli, cognitive control is necessary in order to solve cognitive conflict (Miller and Cohen, 2001; Braver et al., 2002). Cognitive conflict would appear in situations with simultaneous presence of strong and weak stimulus elements, where the strong element causes a perceptual bottom-up response tendency, and where there is a top-down urge to process the weak element. Cognitive control is thus the use of cognitive resources to successfully manage cognitive conflict and to resolve stimulus or instruction interference (Hugdahl et al., 2009a,b). A classic example of a cognitive conflict that would require cognitive control as defined above is the Stroop incongruent color task (Stroop, 1935), where the strong semantic properties of the color word stimulus are in conflict with the weak ink color the word is written in. Another situation fitting this definition is the so called “forced-attention” dichotic listening task where the strong right ear stimulus is in conflict with the instruction to process the weak left ear stimulus in the dichotic listening situation (Hugdahl et al., 2009a).

The ability to generate a correct inhibitory response is present in infancy; however, the consistency of inhibitory control continues to improve throughout childhood and adolescence as evidenced across a range of inhibitory tasks (Luna et al., 2010, 2013) before declining with age (Healey et al., 2008). The brain circuitry supporting adult levels of inhibitory control centers on the prefrontal cortex (PFC), within which the ventral prefrontal cortex (VPFC) is proposed to play a primary role. Imaging studies of frontal gray matter volume reveal distinctly non-linear changes during development, with peak volume arising around 11–12 years of age (being somewhat earlier in females), followed by a rapid decline during adolescence (Gogtay and Thompson, 2010) and further decreases in volume in older adulthood (Elderkin-Thompson et al., 2009). In conjunction with these changes, recent evidence indicates that inhibitory processing in different subdivisions of PFC exhibits different operating characteristics before and after the onset of puberty (Luna et al., 2010). Similarly, environmental influences in these distinct stages of development appear to have different ramifications for frontal/inhibitory functioning (Hackman and Farah, 2009). In sum, this body of knowledge concerning the normative processes underlying cognitive (inhibitory) control provides a template for understanding inhibitory control impairments and the emergence of AH, as well as yielding clues about co-occurring emotional and behavioral disorders.

### Units of analysis: self-report

At the level of self-report (see Table 2) there has been a recent surge of interest in comparing the phenomenology of AH across different groups. Popular symptom assessment tools, such as the Psychotic Symptom Rating Scales (Haddock et al., 1999), include specific information about perceived control of AH (e.g., Can you dismiss or bring on your voices?); though a potential drawback of such questions is that they may index both the ability to suppress along with other forms of control. Nonetheless, this type of structured interview data shows that perceived control over AH is significantly lower in patients with psychosis or borderline personality disorder compared to healthy voice hearers (Jessop et al., 2008; Daalman et al., 2011), but is similar in patients with dissociative identity disorder and schizophrenia (Dorahy et al., 2009). Moreover, patients with schizophrenia and schizoaffective disorder report that they can differentiate their own verbal thoughts from AH (at least in part) on the basis of this sense of control (Hoffman et al., 2008). A notable gap in this literature concerns the lack of detailed phenomenology of hallucinatory experience in older adults; consequently it is still unclear how AH in younger and older adults compare. Consequently, a systematic comparison of self-reported ability to suppress hallucinations across a much broader range of diagnostic and age groups is now warranted. However, though helpful in yielding a deeper insight into voice hearers' experiences, subjective report of controllability is silent on the nature of the mechanisms involved in AH and does not fulfill the need for an objective biomarker or cognitive marker which could provide the foundation for a new diagnostic system for mental illness (Insel, 2013).

**Table 2:** **Summary of key findings on cognitive/inhibitory control impairments associated with hallucinations using the RDoC matrix**.

**COGNITIVE SYSTEMS DOMAIN**							
**Genes**	**Molecules**	**Cells**	**Circuits**	**Physiology**	**Behavior**	**Self-report**	**Paradigms**
**COMPONENT PROCESS: INHIBITION OR SUPPRESSION**							
Heritability estimates for striatal and cortical DA	Increased striatal DA synthesis capacity	Changes in OFC density and/or morphology	Superordinate PFC-cingulate-parietal-subcortical system	ERP 200–300 ms	Inhibitory difficulties or dysfunction	Diminished sense of control, e.g., PSYRATS Item 11 (Controllability of “voices”).	Repeated continuous recognition memory tasks
				Decreased neuronal activation to real voices in patients with AH			
	Hyperexcitation of Glutamate				False alarm rates; reality confusion		
			Posterior medial OFC—sub-cortical loop				Forced-attention dichotic listening task
	Hypoexcitation of GABA			Failure of inhibition and suppression of “voices”	Ability to report weak, left-ear stimulus	Degree of real-time control (iDichotic: iPhone app)	
			Inferior frontal gyrus—anterior cingulate link				
					Focused attention on “real” external voices		

*Abbreviations: AH, auditory hallucination; DA, dopamine; GABA, gamma-Aminobutyric acid; OFC, orbitofrontal cortex; PFC, prefrontal cortex; ERP, event related potential; PSYRATS, Psychotic Symptom Rating Scale*.

Adding to the problem of self-reports is the use of structured interview scales, like the Positive and Negative Syndrome Scale (PANSS: Kay et al., 1987), where data from several dimensions are merged into a common score (1–7) given by the clinician. Consequently, we are currently working on an Smartphone-based “app” where the patient uses the built-in slider to indicate separable aspects of the experience including: the degree to which they feel that the “voices” are coming from the inside or outside of the head (localization dimension); are negative or positive in valence (emotional dimension); and how much cognitive control they have over the “voices” (cognitive dimension). The data are stored in the iPhone/iPod for later retrieval. Such a data-driven approach to quantification of AHs has several advantages over more traditional approaches; it is patient-driven rather than therapist-driven, it is anchored in every-day life situations rather than the confines of a therapist-office, and it allows for data on day-to-day variations in AH parameters not possible to obtain with traditional interview scales. Whilst this is a promising start, there is a need for developing other new approaches to data collection with regard to frequency, intensity and content of AHs.

### Units of analysis: behavior

In contrast, at the behavioral level of analysis considerable progress has been made in identifying the cognitive processes involved in AH, across diagnostic boundaries (Badcock and Hugdahl, 2012a; Waters et al., 2012). Importantly, these studies indicate that AH in schizophrenia and in non-schizophrenia groups are associated with a specific type of impairment in cognitive (rather than motor) inhibition (see Fukushima et al., 1990; for a review Badcock and Hugdahl, 2012b) For example, Badcock and colleagues (Waters et al., 2003; Badcock et al., 2005; Paulik et al., 2007) have examined an inhibitory control process associated with AH in schizophrenia, and in non-clinical hallucinators using a repeated continuous recognition memory task, known as the Inhibition of Currently Irrelevant Memories task (ICIM; originally developed by Schnider, 2003). This task involves the presentation of two (or more) sequences of the same set of pictures, shown in a different order each time. In each run, participants have to indicate picture recurrences *within that run*, irrespective of their presentation in previous runs. So, familiarity supports correct performance on the first run, but in subsequent runs all pictures seem familiar: consequently, accurate performance in the second and later runs depends on the ability to inhibit responses (memories) that are irrelevant in the current sequence. In fact healthy individuals typically produce few errors (false alarms) on this type of task. Though somewhat similar to other source memory tasks which involve knowing which episode in the past a memory refers to, the ICIM task (and its variants) specifically involve “reality filtering,” that is, the ability to distinguish whether a memory pertains to current reality or not (reviewed in Schnider, 2013).

**Figure 1:**
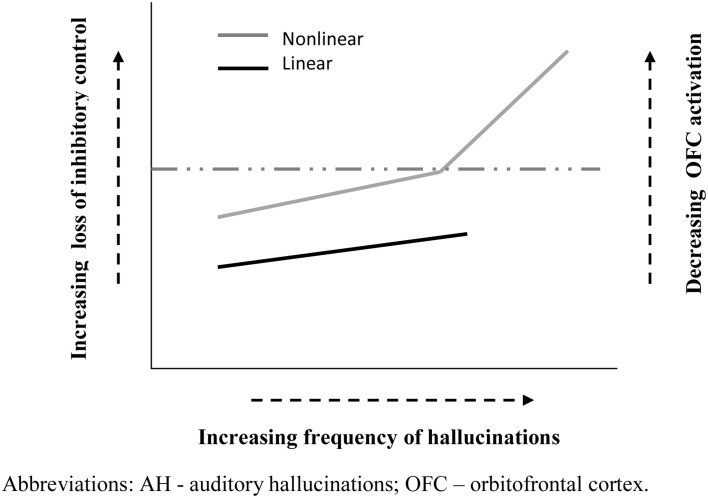
**Schematic representation of possible functional relationships between inhibitory control and hallucination frequency**. Solid black line (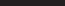) depicts a roughly linear association between inhibition difficulty and AH frequency. Solid gray line (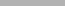) indicates a potentially non-linear relationship between inhibition and AH frequency, characterized by a critical “tipping point” (dashed line) in inhibitory dysfunction, beyond which the frequency of hallucinations rises markedly. Right vertical axis illustrates the link with the degree of OFC activation, which influences the emotional valence of participants' response (details in text).

Applying this task to patients with schizophrenia Waters and colleagues showed that the frequency of AH (but not other symptoms) was associated with an increasing number of false alarms from run to run, signaling an impairment of inhibitory control (Waters et al., 2003). Furthermore, patients with current AH made significantly more false alarms on this task than non-hallucinating patients, whose performance was not significantly different than healthy controls (Badcock et al., 2005). Consequently, this failure to suppress currently irrelevant memories does not appear to be a general feature of schizophrenia, rather it seem to be specifically associated with AH. In other words, hallucinations may come about (in part) when poor inhibitory control allows representations in memory to intrude into current events^3^ and become confused with ongoing reality. Extending these findings beyond the diagnostic boundary of schizophrenia, Paulik and colleagues showed a similar, though somewhat milder, impairment of inhibitory control on the ICIM task in a group of healthy young adults predisposed to hallucinations, suggesting a continuity of mechanisms in clinical and non-clinical hallucinators (Paulik et al., 2007). Furthermore, follow-up studies indicated that the critical component underlying poor performance involves a difficulty intentionally suppressing unwanted cognitions already held in working memory (Paulik et al., 2008). Therefore, one possibility may be that a gradient in the severity of inhibitory control contributes to the greater frequency of hallucinations in psychosis compared to healthy voice hearers; though future research needs to show if this is a truly linear or a non-linear relationship (see Figure 1). Consequently, this specific type of inhibitory control might serve as a cognitive marker of risk for AH and more stringent evidence testing this proposal is now required. Of note, new findings from Badcock's group are encouraging in this regard. This latest data shows that students with high scores on the Hypomanic Personality Scale (Meads and Bentall, 2008), indexing risk for bipolar disorder, are more prone to hallucinate and produce significantly more false alarms on the ICIM task compared to those with low scores.

Clearly, future research needs to extend this approach to investigate a much broader range of clinical and non-clinical groups with AH. In particular, it is clear that the prevalence of hallucinations increases in older adults (aged 60 years plus) (Grimby, 1998; Turvey et al., 2001) whilst inhibitory efficiency declines, yet the direct association between these factors has never been assessed. Furthermore, inhibitory dysfunction is a prominent feature in a variety of typically older patient groups, such as Parkinson's disease (Gurvich et al., 2007) and Alzheimer's disease (Amieva et al., 2004), in which hallucinations are also often reported (Scarmeas et al., 2005; Diederich et al., 2009). It would, therefore, be useful to examine the association between inhibitory control and hallucinatory experience across all of these groups using the same experimental task, in order to determine if they share a common mechanistic basis.

Since cognitive control and inhibition should be independent of sensory modalities, impairments should also be observed for auditory tasks. Hugdahl et al. (2009a,b) have used a variant of the dichotic listening task, both in normal adults and in patients with schizophrenia, to study the ability to inhibit a strong auditory stimulus being presented in one ear simultaneously with a weak stimulus presented in the other ear. The instruction is to focus attention on the weak stimulus and report it in the presence of the strong stimulus, which would require inhibitory control in order to resolve the cognitive conflict caused by the simultaneous presence of the strong and weak stimuli. In several studies, it has been shown that patients with schizophrenia in general are impaired compared to healthy controls to inhibit the strong right ear stimulus and report the weak left ear stimulus (Løberg et al., 1999), and negative correlations between the score on the PANSS hallucination item and the ability for inhibitory control in order to resolve the cognitive conflict (Hugdahl et al., 2013). The data from the dichotic listening task has also revealed that individual differences between patients with schizophrenia are larger than corresponding differences for healthy controls, something that could be related to the specific role played by AH in interfering with cognitive control and inhibition.

**Figure 2:**
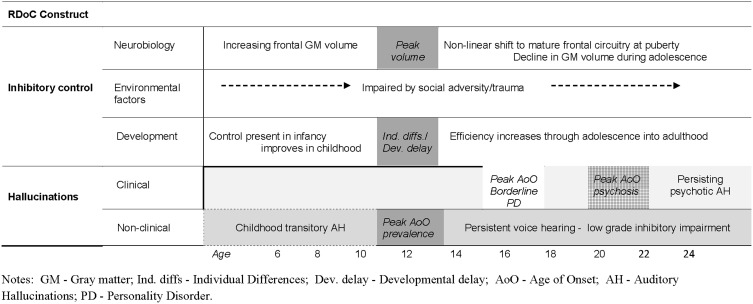
**Schematic illustration of potentially separate pathways to clinical and non-clinical hallucinations linked to the dynamics of inhibitory development**.

The behavioral results described so far emphasize the importance of *individual differences* in inhibitory control in AH. In contrast, the role of *age-related differences* in inhibitory efficiency have received scant attention, yet are also likely to be important in the developmental expression of AH (such as the average age of onset of AH) in clinical and non-clinical groups. Population-based studies show that AH are relatively common in childhood (Bartels-Velthuis et al., 2010; Laurens et al., 2012), but typically do not last (De Loore et al., 2011). For example, Bartels-Velthuis and colleagues reported that in young hallucinators (7–8 years) approximately three quarters had discontinued by 13 years (Bartels-Velthuis et al., 2011)—an age when frontal cortical systems have usually shifted to a mature pattern of functioning. Thus, the vast majority of hallucinations in childhood may reflect—in part—normal fluctuations in development of inhibitory control, which eventually self-correct. For others, inhibitory skills may continue to lag behind their peers and the tendency to hallucinate persists into adulthood, transitioning to psychosis in many—though not all (Fusar-Poli et al., 2013; Linscott and van Os, 2013). Critically, the timing of onset of AH differs in these groups: for example, in schizophrenia/schizoaffective disorder, borderline personality disorder and healthy (non-patient) hallucinators the average age of onset is around 20, 16, and 13 years, respectively, (Daalman et al., 2011; Slotema et al., 2012). A major issue for the next generation of studies will be to ascertain how the dynamics of inhibitory control before and after puberty contributes to the variation in onset and persistence of AH across diagnostic groups. Moreover, these different developmental trajectories undermine the notion of a single continuum underlying clinical and non-clinical AH, in favor of separate developmental trajectories (see Figure 2). Indeed, the growing body of evidence of differences between psychotic and non-psychotic hallucinations—in the details of their form and function—is more consistent with the presence of at least two distinct continua (Daalman et al., 2011; Badcock and Hugdahl, 2012a; Linscott and van Os, 2013).

### Units of analysis: cells, circuits, and physiology

The physiological mechanism underlying the inability to intentionally suppress currently irrelevant memories appears to depend on the orbitofrontal cortex (OFC) and related sub-cortical circuits (see Schnider, 2013). For example, numerous previous studies have documented a reality filtering deficit in patients with spontaneous confabulations^4^ due to damage in posterior medial OFC or regions directly connected to it. These patients produce a steep increase in false positive responses on the ICIM task across runs, along with an electrophysiological signature of deficient transient inhibition at 200–300 ms (see Schnider, 2003, 2008; Whelan et al., 2012). Conversely, healthy individuals who perform the task well show distinct activation of OFC (Schnider et al., 2000). These observations suggest that OFC provides a very particular type of memory control, signaling whether an activated memory relates to ongoing reality or not. The absence of evidence directly linking OFC dysfunction and hallucinations across diagnostic categories is an obvious gap in the literature. However, the OFC is a particular region of interest in psychotic disorders and in ultra-high risk cohorts (who often report brief or attenuated AH) believed to be at increased risk for psychosis (Jung et al., 2012; Kubota et al., 2012; Bartholomeusz et al., 2013). For example, progressive gray matter loss in OFC and temporal regions predates the onset of psychosis in ultra-high risk groups but not in those who do not progress to illness, (Gogtay et al., 2011) and may be a heritable component of both schizophrenia and bipolar disorder (Moran et al., 2013). These findings would be compatible with a role for OFC as a neuro-anatomical marker of risk for AH as psychosis is developing, but this proposal is yet to be validated.

The OFC is also known to be involved in a range other functions, including social and emotional cognition (Nestor et al., 2013), which may provide new avenues for understanding AH. For example, the degree of activation of OFC is correlated with the valence (pleasure/displeasure) of participants' emotional response: as activity decreases the unpleasantness experienced increases and pleasantness decreases (Wilson-Mendenhall et al., 2013). Thus, underlying differences in OFC activation could contribute to the prominent differences in emotional valence of AH in clinical (predominantly negative: less OFC activation) and non-clinical (predominantly positive: more OFC activation) groups (Daalman et al., 2011; see Figure 1). In fact, if this proposal is correct then significant differences in age of onset, frequency and valence of AH in psychotic and healthy voice hearers may share a common functional basis, linked to the degree of impairment in OFC circuitry. Other areas and networks which are critical for cognitive control as a way of resolving cognitive conflict include the dorsolateral PFC, and the anterior cingulate cortex (ACC) (Niendam et al., 2012). For example, Hugdahl's group has shown that in healthy young and old subjects the inhibitory control seen in the forced-attention dichotic listening task is mediated by the inferior frontal gyrus and anterior cingulate gyrus in the PFC (Thomsen et al., 2004). These brain regions have also been implicated in failure of inhibitory control in schizophrenia patients (Hugdahl et al., 2009b), and in particular the failure to direct attention control away from the “voices” and toward stimuli in the surrounding environment.

### Units of analysis: molecules and genes

Emerging evidence from molecular genetics points to several candidate genes associated with an increased risk for hallucinations in schizophrenia or schizoaffective disorder (Sun et al., 2012), implicating multiple neurotransmitter systems, though their specific contribution to orbitofrontal reality filtering remains largely uncharted territory. The exception in this literature is that the ability to suppress currently irrelevant memories, as measured with the ICIM task, is known to be modulated by the neurotransmitter dopamine (Pihan et al., 2004). For example, healthy individuals in a hyper-dopaminergic state (induced by L-dopa) have been shown to produce a specific increase in false alarms on a challenging version of the ICIM test compared to when they received a dopamine antagonist (risperidone) (Schnider et al., 2010). Importantly, the detection of picture repetitions was not significantly influenced by L-dopa, which makes a general failure of behavioral inhibition unlikely. Indeed, Schnider (2013) has suggested that in healthy subjects a specific process may be involved in which OFC activity transiently inhibits subcortical dopaminergic neurons, signaling that a memory doesn't relate to the present reality. These observations are intriguing in view of the long-held belief that dopaminergic dysfunction is involved in the development of psychosis (Laruelle and Abi-Dargham, 1999). In fact, current theories suggest that striatal hyper-dopaminergia is centrally involved in the emergence of positive symptoms such as AH (Howes and Kapur, 2009) and elevated striatal dopamine synthesis capacity is consistently reported in those at ultra-high risk for psychosis (Howes et al., 2009, 2011; Egerton et al., 2013). As yet, it is unknown if similar elevations occur in other diagnostic groups experiencing AH. However, recent data shows that dopamine synthesis capacity is *not* significantly different in healthy individuals with AH compared to non-hallucinating controls (Howes et al., 2013), thus the endogenous neurobiological determinants of AH seem to differ in those on a trajectory to psychosis vs. healthy voice hearers.

In addition to a hyper-dopaminergia as contributing to positive symptoms, it is possible that both glutamate and gamma-Aminobutyric acid (GABA) are involved in the elicitation of AH and the failure of cognitive control functions to inhibit or control the “voices” once they occur, respectively. Hyper-excitation of glutamate receptors in temporal lobe areas could be mediating findings of increased neuronal activation in these areas during states of hallucinations, at least in patients with schizophrenia (see Silbersweig et al., 1995; Dierks et al., 1999; Jardri et al., 2011; Kompus et al., 2011 for studies showing increased activation in relation to AHs), possibly causing the perception of someone speaking to the patient. It is often forgotten that glutamate receptors are the most widely distributed receptors in the cortex (Buzsaki et al., 2007), and glutamate being the primary excitatory transmitter. This is in contrast to dopamine receptors which are relatively few in the PFC compared to the striatum and basal ganglia. Similarly, it could be hypothesized that hypo-excitation of GABA receptors, being the major inhibitory transmitter, in prefrontal areas could be a mediating factor behind a failure of inhibitory control in AH patients with psychosis. Consequently, we suggest the glutamate/GABA balance in temporal and frontal lobe areas may be disturbed in schizophrenia, and that the relative imbalance of glutamate and GABA activity may be mediating both the perceptual quality of AHs and the reduced resources for inhibitory control seen in these patients. Glutamate and GABA concentrations can be measured on a voxel-basis in the brain with MR spectroscopy, and although quantification of these metabolites, and in particular GABA, are currently a challenge, promising new ways how to circumvent these measurement problems are under way in several labs around the world (see Puts and Edden, 2012; Mullins et al., 2014). A glutamate hypothesis behind some of the pathophysiology seen in schizophrenia is not new (see Coyle, 2006—where abnormal functioning of glutamate receptors, and the interaction with GABA receptor inter-neurons, has been suggested to be involved in the pathophysiology of schizophrenia, and contribute to negative symptoms). We now suggest an extension of the glutamate hypothesis for schizophrenia by suggesting that the glutamate/GABA axis may play a significant role also for positive symptoms and in particular for AH (cf. Allen et al., 2012).

In drilling down through the various levels of analysis in the RDoC matrix it is easy to succumb to an exclusively biological and unidirectional explanatory model of AH (e.g., genes→OFC→inhibitory control→AH). So it's important that each unit of analysis is also viewed from an environmental lens. For example, there is a substantial body of evidence linking childhood trauma and social adversity with the emergence of AH (e.g., in psychosis and non-clinical hallucinators, Bartels-Velthuis et al., 2012; Bentall et al., 2012; Daalman et al., 2012; bipolar disorder, Hammersley et al., 2003; borderline PD, Schroeder et al., 2013). The mechanisms involved are still far from clear and undoubtedly complex. However, several recent lines of evidence suggest, for example, that striatal dopamine function is adaptive to individual environmental stressors (Martinez et al., 2010; Stokes et al., 2013) and, therefore, provides at least one plausible explanation for *how* trauma increases the risk for AH across diagnostic boundaries. Thus, based on the available data, exposure to significant social stressors should be associated with elevated DA synthesis, which in turn should lead to greater difficulty in reality filtering (i.e., inhibitory control) and a corresponding increase in the tendency to hallucinate—regardless of diagnosis. Clearly, there may be other causal pathways involved (Gracie et al., 2007; Varese et al., 2012), but this specific theoretical proposal, illustrating the potential power of the RDoC framework, is readily testable and requires further investigation. In sum, by systematically integrating environmental and biological perspectives we may get a more complete understanding of the mechanisms underlying AH in clinical and non-clinical groups.

### Units of analysis: paradigms

A dimensional approach in the sense advocated by the RDoC approach would ideally allow for assessment and quantification of different cognitive domains within the same assessment paradigm. This is typically not done, and for good reasons since different cognitive constructs, e.g., perception, attention, cognitive control, traditionally require different paradigms or tests when assessing patients and healthy individuals. However, the ICM task allows for assessment of memory as well as intentional inhibition within the same task. Furthermore, since the component processes critical to AH are becoming clear, the next step should be to delineate its psychometric properties and optimal configuration (e.g., number of runs, target to distracter ratio) in order to assist its translation into clinical practice.

Similarly, a variant of the dichotic listening paradigm, originally labeled “forced-attention” dichotic listening by Hugdahl and Andersson (1986) allows for the successive assessment of perceptual, attention and cognitive control abilities within the same paradigm. Originally an experimental paradigm for the assessment of cerebral localization of speech perception, the forced-attention dichotic listening paradigm has become a tool for clinical assessment of a wide range of psychiatric and neurological disorders (see Hugdahl et al., 2009a,b for an overview), and would nicely fit the requirements for an assessment paradigm that cuts across cognitive domains as indicated in the RDoC. In two recent papers, Hugdahl et al. (2012, 2013) have shown that patients with AH clearly deviate from healthy controls on the three cognitive constructs—perception, attention, and cognitive control—and that this paradigm could be a valuable tool for assessing AH in both psychotic and non-psychotic individuals hearing “voices.” There are several advantages with being able to study different cognitive constructs with a single paradigm, which is opposite to the more traditional approach of studying a single construct with different paradigms. First of all a single paradigm allows for within-construct comparisons on the same individual, i.e., differences in degree of impairment between the implicated cognitive constructs can be statistically evaluated on an individual level. Second, it allows for statistical evaluation of interaction effects between constructs and patients vs. healthy controls. Third, it allows for tight experimental control, since all stimulus parameters stay constant across the different conditions, only the instructions differ, inducing different cognitive constructs. The idea of developing novel paradigms, or utilizing existing paradigms in novel applications for the study of AH, and also other aspects of mental disorders, that would follow from an RDoC approach is an interesting and promising endeavor that is to be encouraged in future research.

## Comorbidity and divergent trajectories

An additional advantage that emerges from the RDoC approach is the potential to account for co-occurring symptoms routinely associated with AH in community and clinic settings. For example, a range of studies have shown that hallucinations during childhood often present with concurrent emotional (e.g., anxiety, depression) and behavioral (e.g., conduct problems, inattention) difficulties (Askenazy et al., 2007; Armando et al., 2010; Laurens et al., 2012), and those that persist are associated with an increased risk for both internalizing and externalizing disorders later in adolescence (Downs et al., 2013; Kelleher et al., 2013) as well as social dysfunction, affective disorder, substance misuse and psychosis in adulthood (Dhossche et al., 2002; Rossler et al., 2007; De Loore et al., 2011; van Rossum et al., 2011). This pattern of comorbidity and divergent trajectories is consistent with the idea that psychotic symptoms index risk for a wide range of psychopathological outcomes, not limited to psychosis (Rossler et al., 2011; Kelleher et al., 2012a,b; Fusar-Poli et al., 2013), and points to some shared or common deficits in cognitive control. Current evidence shows, for example, that in healthy individuals the OFC plays a role in emotional processing, complex decision making and adaptive social behavior (Kringelbach, 2005; Farrow et al., 2011) whilst impaired inhibitory control and hypo-activation of OFC, typical of AH, have been linked with adolescent impulsivity and initiation of drug use (Whelan et al., 2012) as well as oppositional defiant disorder and conduct disorder (Matthys et al., 2013). We suggest that whilst several previous studies have documented the range of emotional and behavioral difficulties that occur alongside AH they have largely failed to unravel the etiological processes involved. However, by adopting the forced-attention dichotic listening task and/or the ICIM task we may now begin to explore the mechanisms underlying the complex symptom presentation and divergent trajectories associated with AH.

## Treatment implications

Although the RDoC initiative is a classification system aimed at research (Insel et al., 2010), its different domains and dimensions may have some varied implications for treatment. Before discussing such implications, we would like to make a distinction regarding what is meant by “treatment” in general, since this is typically confused in the literature. Our argument is that depending on how one looks at “treatment,” the RDoC approach may be relatively more applicable to one level of treatment than another. We would like to introduce a three-level approach to the meaning of “treatment”: (1) in a classic and every-day meaning of the word, “treatment” is used in the sense of “curing” the patient. For schizophrenia this would mean the abolishment of symptoms, restoration of normal cognitive and emotional functioning, normal social and interpersonal behavior. This usage is synonymous with pharmacological treatment. (2) A second meaning is what is typically seen as the purview of psychotherapy, which aims at changing the mind-setting of the patient, to induce a different “strategy” of how to think of symptoms, other people, the environment etc. Here the patient is not “cured,” but adopts a new cognitive strategy of how to meet psychological challenges. (3) A third meaning of the word is in the sense of a “training” routine, which aims at changing a specific behavioral or cognitive function, e.g., to train how to learn to shift attention away from hallucinated “voices” and to external stimuli whenever the “voices” occur: here the patient is not cured, is not taught a new general strategy, but learns how to cope with a specific symptom, whenever it occurs. We would like to suggest that focus on the different RDoC may have different treatment implications, operating over different time scales. For example, focus on the “Arousal/modulatory systems,” including also biological rhythms and sleep-wake cycles, might have immediate implications for pharmacological treatment in the classic sense of curing the patient. Focus on “Systems for social processes” containing affiliation and attachment, social communication, and the understanding of others would have longer term implications for a psychotherapy approach to treatment, while focus on the “Cognitive systems,” like attention, working memory etc. will have both short and long-term implications for a training approach to treatment. These examples are clearly not exhaustive but are included to illustrate the flexibility that the RDoC approach to psychiatric diagnosis allows.

Turning now to our specific focus on cognitive control we argue that a better understanding of the mechanisms involved in inhibitory processing may have clinical implications for the treatment of AH through the development of new cognitive training routines where patients can learn how to overcome loss of inhibitory control by being trained to focus attention of the weaker surrounding stimuli rather than the stronger inner “voices.” For example, Soveri et al. (2013) have already shown how training to re-focus attention to the weaker sound in the dichotic listening situation can be established after 3–4 weeks of daily training. However, caution should be invoked when interpreting these findings since this study was performed on healthy individuals and such findings cannot be automatically generalized to patients. However, recent attempts to apply the same paradigm to patients experiencing AH have provided some promising results. These direct clinical attempts, using an iPod Touch version of the paradigm, involve patients being trained in their home environment for 3–4 weeks under guidance, and then being given the device to use whenever they feel the “voices” are becoming overwhelming. Although not all patients have been equally successful in increasing their ability to re-focus attention and exert cognitive control, most of the patients tested so far (*N* = 15) have reported that “*training has helped me to start fighting back when the voices command me, not just be a passive follower of what they say,” and “I feel more confident of myself and that I can withstand the voices”* which are quotations from two patients after 4 weeks of training with the iPod Touch app. Clearly this preliminary evidence requires experimental validation, but these early clinical reports provide essential information about how patients actually experience a remediation attempt and are too often ignored in clinical research. Thus, a simple smart phone device can open up new avenues for cognitive training, which may have direct clinical utility. Similarly, a glutamate/GABA perspective on AH may also have clinical implications since current drug treatments primarily target dopamine D_1/2_ receptors (Howes et al., 2012), and the development of new drug targets could be beneficial for the amelioration of AH symptoms as well. In sum, we suggest that similar novel treatment approaches may be derived from an RDoC approach, and should be explored in future research and clinical practice.

## Concluding remarks, future directions—vertical synergy

The RDoC project takes a fundamentally dimensional approach to conceptualizing mental illness and therefore has much in common with existing continuum models of AH. Our review significantly extends this previous work by encouraging a more integrated understanding of the biological, cognitive, and social mechanisms of inhibitory control involved in AH, framed within an explicitly developmental perspective. It also provides new insights into the complex mix of other non-psychotic symptoms that often co-occur with AH, and suggests a stratified way of thinking about treatment. However, in completing the RDoC matrix it is clear that a number of gaps and shortcomings in the available data on AH remain. Most importantly, research on AH in psychosis has tended to overshadow studies of the experience of hallucinations in other mental disorders, which deserve a greater focus by the research community. Similarly, whilst behavioral and physiological work on AH has made significant advances we note (Table 2) the dearth of evidence linking the cellular level of analysis to AH and inhibitory control, which warrants further investigation (Johnsen et al., 2013), as well as far too little attention to the impact of environmental/social factors on each separate unit of functioning. Indeed a challenge for future research will be to examine multiple units of analysis simultaneously, to probe the behavioral and psychological mechanisms of inhibitory control that drive the onset and persistence/severity of AH.

We are aware that the focus in this article is limited to only a single domain of [cognitive] functioning, and it is clear that future research will need to determine how dysregulation in this domain interacts with other domains of functioning known to be important in AH (such as affective prosody deficits in the Social Process domain) or for effective cognitive performance in general (such as circadian variability from the Arousal/regulatory systems domain). An important implication arising from this analysis is that mixing participants with and without AH within a single study means that you will have groups that draw on different etiological mechanisms. Future research designs will benefit from linking phenomenological, neuronal and cognitive data (rows × columns) to form more homogenous clusters of hallucinatory experiences that can then be used to develop individually tailored treatments (Stephane, 2013). We would like to end this review of possible implications for research of an RDoC approach by providing an example of a comprehensive research agenda focused on AH that attempts to track the understanding of the underlying mechanisms in AH from the molecular to the clinical levels of explanation. We have labeled such an approach “vertical synergy,” in the sense of utilizing knowledge from different levels of understanding in a vertical perspective, from phenomenology to molecules. A vertical synergy agenda is inspired by the ideas in the RDoC approach and represents a “rows × columns” matrix for advancing our knowledge of one of the most severe symptoms, AH, in one of the most severe mental disorders, schizophrenia. Starting at the top, most empirical evidence points to the fact that the phenomenology of AH involves a subjective experience of hearing a “voice.” This means that at the second level of explanation—the cognitive level—AH involves a perceptual quality, and in particular the quality of a speech percept since the subjective experience is typically of a person “speaking to the patient.” The perceptual nature of AH has been evidenced in the studies by Green et al. (1994), Woodruff et al. (1997), and Hugdahl et al. (2012). Granted a speech perception explanation at the cognitive level of analysis, the next question is: “what could be causing such an experience in the absence of a physical stimulus, at the neuronal level?” A reasonable first hypothesis would be to look for neuronal abnormality in brain areas connected with speech perception in the temporal lobes, moving down to a circuitry, or systems, level of explanation. Several studies have revealed faulty neuronal networks involving the temporal lobes in hallucinating patients, using EEG, PET, and fMRI (Silbersweig et al., 1995; Dierks et al., 1999; Ford et al., 2001; Hugdahl et al., 2008; see also meta-analyses in Jardri et al., 2011 and Kompus et al., 2011) confirming abnormality in especially left temporal lobe areas during AHs, which are hyper-activated during AH episodes. At the same time, Hugdahl et al. (2013) showed that the ability to suppress an external stimulus is negatively correlated with the frequency of AH, pointing to a corresponding hypo-activation of prefrontal brain areas, which could explain why AHs are not inhibited once perceived. All this leads to the next level of explanation, i.e., which transmitters and receptors are dysfunctional?- causing neuronal abnormality at the circuitry level, which in turn gives rise to a virtual perceptual experience that has the phenomenological, or subjective, quality of a person “speaking” to the individual, and why this is not suppressed or inhibited. Several hypotheses have been advanced that involve both the dopaminergic and glutamatergic transmitter systems. From the hypothesis advocated by Kapur and Mamo (2003) that dopaminergic transmission is dys-regulated in schizophrenia, leading to an abnormal salience to external and internal stimuli, it could be further hypothesized that AH result from an abnormal salience to inner stimuli that occur spontaneously (see also Johnsen et al., 2013). Such a hypothesis can, however not explain why the “voices” are not inhibited, only how they are initiated. A more recent, untested, proposal is therefore that glutamaterigic hyper-activity may be directly or indirectly involved in the elicitation of AH and that these are not cognitively inhibited owing to GABAergic hypo-activity in PFC (Johnsen et al., 2013). This is a challenging hypothesis, that could be tested with MR spectroscopy from voxels in the temporal and frontal lobes, respectively, which could explain both the spontaneous excitatory initiation due to glutamate activity, and subsequent failure of inhibition due to deficient GABA activity. An RDoC approach would also require an attempt to explain AH at the molecular, genetic level of analysis. However, as stated by Sanjuan et al. (2013), “*although a large number of studies have examined the influence of environmental risk factors…for hallucinations…the molecular genetic predispositions has received little attention”* (p. 235). This also holds for a dimensional approach to hallucinations, in that no molecular study exists of the genetic basis for “hearing voices” in non-psychotic individuals. A few studies exist that have linked genetic influences on glutamate, GABA and dopamine effects on neurons in a thalamo-cortical network, although the results are far from conclusive (Behrendt and Young, 2004). Thus, more research is needed to reveal the underlying molecular architecture behind AH in both clinical and non-clinical populations. Because AH manifest itself as such powerful behaviors, influencing the entire cognitive and emotional set-up of an individual it is reasonable to assume that such pervasive behavioral effects must have a biological cause.

### Conflict of interest statement

The authors declare that the research was conducted in the absence of any commercial or financial relationships that could be construed as a potential conflict of interest.

## Footnotes

^1^For full description see: http://www.nimh.nih.gov/research-funding/rdoc/index.shtml

^2^We refer to voices and “voices” to distinguish the perception of voices in the presence or absence of an external stimulus, respectively.

^3^Such intrusions are not limited to entire episodes from memory (as in traumatic replay of whole scenes) but may include intrusion of individual components of stored representations (such as words, voices etc).

^4^These clinical studies also serve as a reminder that dysfunctional inhibition is not solely associated with AH.

## References

[B1] Allen P. Modinos G. Hubl D. Shields G. Cachia G. Jardri R. (2012). Neuroimaging auditory hallucinations in schizophrenia: from neuroanatomy to neurochemistry and beyond. Schizophr. Bull. 38, 695–703 10.1093/schbul/sbs066 22535906 PMC3406523

[B2] Amieva H. Phillips L. H. DellaSala S. Henry J. D. (2004). Inhibitory functioning in alzheimer's disease: a review. Brain 127, 949–964 10.1093/brain/awh045 14645147

[B3] Armando M. Nelson B. Yung A. R. Ross M. Birchwood M. Girardi P. (2010). Psychotic-like experiences and correlation with distress and depressive symptoms in a community sample of adolescents and young adults. Schizophr.Res.119, 258–265 10.1016/j.schres.2010.03.001 20347272

[B4] Askenazy F. L. Lestideau K. Meynadier A. Dor E. Myquel M. Lecrubier Y. (2007). Auditory hallucinations in pre-pubertal children. A one year follow-up, preliminary findings. Eur. Child Adolesc.Psychiatry 16, 411–415 10.1007/s00787-006-0577-9 17468968

[B5] Badcock J. C. (2010). The cognitive neuropsychology of auditory hallucinations: a parallel auditory pathways framework. Schizophr.Bull. 36, 576–584 10.1093/schbul/sbn128 18835839 PMC2879695

[B6] Badcock J. C. Chhabra S. (2013). Voices to reckon with: perceptions of voice identity in clinical and non-clinical voice hearers. Front. Hum. Neurosci.7:114 10.3389/fnhum.2013.00114 23565088 PMC3615181

[B7] Badcock J. C. Hugdahl K. (2012a). Cognitive mechanisms of auditory verbal hallucinations in psychotic and non-psychotic groups. Neurosci. Biobehav. Rev. 36, 431–438 10.1016/j.neubiorev.2011.07.010 21827786

[B8] Badcock J. C. Hugdahl K. (2012b). Examining the continuum model of auditory hallucinations: a review of cognitive mechanisms, in Hallucinations: Research and Practice, ed Blom J. D. Sommer I. E. C. (New York, NY: Springer Science + Business Media), 317–330

[B9] Badcock J. C. Laroi F. Allen P. Diederen K. (2014). Current perspectives on the mechanisms of auditory hallucinations in clinical and non-clinical populations. Frontiers Media, SA 10.3389/978-2-88919-203-8 PMC3831087 24302908

[B10] Badcock J. C. Waters F. A. V. Maybery M. Michie P. T. (2005). Auditory hallucinations: failure to inhibit irrelevant memories. Cogn. Neuropsychiatry 10, 125–136 10.1080/13546800344000363 16571456

[B11] Bartels-Velthuis A. A. Jenner J. A. van de Willige G. van Os J. Wiersma D. (2010). Prevalence and correlates of auditory vocal hallucinations in middle childhood. Br. J. Psychiatry 196, 41–46 10.1192/bjp.bp.109.065953 20044659

[B12] Bartels-Velthuis A. A. van de Willige G. Jenner J. A. van Os J. Wiersma D. (2011). Course of auditory vocal hallucinations in childhood: 5-year follow-up study. Br. J. Psychiatry 199, 296–302 10.1192/bjp.bp.110.086918 21708881

[B13] Bartels-Velthuis A. A. van de Willige G. Jenner J. A. Wiersma D. van Os J. (2012). Auditory hallucinations in childhood: associations with adversity and delusional ideation. Psychol. Med. 42, 583–593 10.1017/S0033291711001590 21861954

[B14] Bartholomeusz C. F. Whittle S. L. Montague A. Ansell B. McGorry P. D. Velakoulis D. (2013). Sulcogyral patterns and morphological abnormalities of the orbitofrontal cortex in psychosis. Prog. Neuropsychopharmacol. Biol. Psychiatry 44(Suppl. C), 168–177 10.1016/j.pnpbp.2013.02.010 23485592

[B15] Behrendt R. P. Young C. (2004). Hallucinations in schizophrenia, sensory impairment, and brain disease: A unifying model. Behav. Brain Sci., 27, 771–787 10.1017/S0140525X04000184 16035402

[B16] Bentall R. P. (1996). At the centre of a science of psychopathology? Characteristics and limitations of cognitive research. Cogn. Neuropsychiatry 1, 265–273 10.1080/135468096396433 16571492

[B17] Bentall R. P. Wickham S. Shevlin M. Varese F. (2012). Do specific early-life adversities lead to specific symptoms of psychosis? A study from the 2007 the adult psychiatric morbidity survey. Schizophr. Bull. 38, 734–740 10.1093/schbul/sbs049 22496540 PMC3406525

[B18] Blom J. D. Sommer I. E. C. (2012). Hallucinations: Research and Practice. New York, NY: Springer Science + Business Media 10.1007/978-1-4614-0959-5

[B20] Braver T. S. Cohen J. D. Barch D. M. (2002). The role of the prefrontal cortex in normal and disordered cognitive control: a cognitive neuroscience perspective, in Principles of Frontal Lobe Function, ed Stuss D. T. Knight R. T. (Oxford: Oxford University Press), 428–448

[B21] Buzsaki G. Kaila M. Raichle M. (2007). Inhibition and brain work. Neuron 56, 771–783 10.1016/j.neuron.2007.11.008 18054855 PMC2266612

[B22] Cole C. (2013). NIMH's new framework for classifying and researching psychopathology. Observer 26, 42 Available online at: http://www.psychologicalscience.org/index.php/publications/observer/2013/july-august-13/nimhs-new-framework-for-classifying-and-researching-psychopathology.html

[B23] Coyle J. T. (2006). Glutamate and schizophrenia: beyond the dopamine hypothesis. Cell. Mol. Neurobiol. 26, 365–384 10.1007/s10571-006-9062-8 16773445 PMC11881825

[B24] Cuthbert B. N. Insel T. R. (2013). Toward the future of psychiatric diagnosis: the seven pillars of RDoC. BMC Med. 11:126 10.1186/1741-7015-11-126 23672542 PMC3653747

[B25] Daalman K. Boks M. P. Diederen K. M. de Weijer A. D. Blom J. D. Kahn R. S. (2011). The same or different? A phenomenological comparison of auditory verbal hallucinations in healthy and psychotic individuals. J. Clin. Psychiatry 72, 320–325 10.4088/JCP.09m05797yel 21450152

[B26] Daalman K. Diederen K. M. Derks E. M. van Lutterveld R. Kahn R. S. Sommer I. E. (2012). Childhood trauma and auditory verbal hallucinations. Psychol. Med. 42, 2475–2484 10.1017/S0033291712000761 22716897

[B27] David A. (2004). The cognitive neuropsychiatry of auditory verbal hallucinations: an overview. Cogn. Neuropsychiatry 9, 107–123 10.1080/13546800344000183 16571577

[B28] de Leede-Smith S. Barkus E. (2013). A comprehensive review of auditory verbal hallucinations: lifetime prevalence, correlates and mechanisms in healthy and clinical individuals. Front. Hum. Neurosci. 7:367 10.3389/fnhum.2013.00367 23882203 PMC3712258

[B29] De Loore E. Gunther N. Drukker M. Feron F. Sabbe B. Deboutte D. (2011). Persistence and outcome of auditory hallucinations in adolescence: a longitudinal general population study of 1800 individuals. Schizophr. Res. 127, 252–256 10.1016/j.schres.2011.01.015 21315559

[B30] Dhossche D. Ferdinand R. Van der Ende J. Hofstra M. B. Verhulst F. (2002). Diagnostic outcome of self-reported hallucinations in a community sample of adolescents. Psychol. Med. 32, 619–627 10.1017/S003329170200555X 12102376

[B31] Diederich N. J. Fénelon G. Stebbins G. Goetz C. G. (2009). Hallucinations in Parkinson disease. Nat. Rev. Neurol. 5, 331–342 10.1038/nrneurol.2009.62 19498436

[B32] Dierks T. Linden D. E. J. Jandl M. Formisano E. Goebel R. Lanfermann H. (1999). Activation of Heschl's gyrus during auditory hallucinations. Neuron 22, 615–621 10.1016/S0896-6273(00)80715-1 10197540

[B33] Dorahy M. J. Shannon C. Seagar L. Corr M. Stewart K. Hanna D. (2009). Auditory hallucinations in dissociative identity disorder and schizophrenia with and without a childhood trauma history: Similarities and differences. J. Nerv. Ment. Dis. 197, 892–898 10.1097/NMD.0b013e3181c299ea 20010024

[B34] Downs J. M. Cullen A. E. Barragan M. Laurens K. (2013). Persisting psychotic-like experiences are associated with both externalising and internalising psychopathology in a longitudinal general population child cohort. Schizophr. Res. 144, 99–104 10.1016/j.schres.2012.12.009 23321428

[B35] Egerton A. Chaddock C. A. Winton-Brown T. T. Bloomfield M. A. Bhattacharyya S. Allen P. (2013). Presynaptic striatal dopamine dysfunction in people at ultra-high risk for psychosis: findings in a second cohort. Biol. Psychiatry 74, 106–112 10.1016/j.biopsych.2012.11.017 23312565

[B36] Elderkin-Thompson V. Hellemann G. Pham D. Kumar A. (2009). Prefrontal brain morphology and executive function in healthy and depressed elderly. Int. J. Geriatr. Psychiatry 24, 459–468 10.1002/gps.2137 18819162

[B37] Farrow T. F. D. Jones S. C. Kaylor-Hughes C. J. Wilkinson I. D. Woodruff P. W. R. Hunter M. D. (2011). Higher of lower? The functional anatomy of perceived allocentric social hierarchies. Neuroimage 57, 1552–1560 10.1016/j.neuroimage.2011.05.069 21664277

[B38] Fisher H. L. Caspi A. Poulton R. Meier M. H. Houts R. Harrington H. (2013). Specificity of childhood psychotic symptoms for predicting schizophrenia by 38 years of age: a birth cohort study. Psychol. Med.10, 1–10 10.1017/S0033291712003091 23302254 PMC3758773

[B39] Ford J. M. Mathalon D. H. Kalba S. Whitfield S. Faustman W. O. Roth W. T. (2001). Cortical responsiveness during inner speech in schizophrenia: an event-related potential study. Am. J. Psychiatry 158, 1914–1916 10.1176/appi.ajp.158.11.1914 11691701

[B40] Ford J. M. Morris S. Hoffman S. I. Waters F. McCarthy-Jones S. Cuthbert B. (in press). Studying hallucinations within the NIMH RDoC framework: report from the 2nd international consortium on hallucinations research. Schizophr. Bull. 10.1093/schbul/sbu011 PMC4141312 24847862

[B41] Freeman D. Dunn G. Garety P. Weinman J. Kuipers E. Fowler D. (2013). Patients' beliefs about the causes, persistence and control of psychotic experiences predict take-up of effective cognitive behaviour therapy for psychosis. Psychol. Med. 43, 269–277 10.1017/S0033291712001225 22781166 PMC3544544

[B42] Fukushima J. Morita N. Fukushima K. Chiba T. Tanaka S. Yamashita I. (1990). Voluntary control of saccadic eye movements in patients with schizophrenic and affective disorders. J. Psychiatr. Res. 24, 9–24 10.1016/0022-3956(90)90021-H 2366215

[B43] Fusar-Poli P. Borgwardt S. Bechdolf A. Addington J. Riecher-Rossler A. Schultze-Lutter F. (2013). The psychosis high-risk state: a comprehensive state-of-the-art review. JAMA Psychiatry 70, 107–120 10.1001/jamapsychiatry.2013.269 23165428 PMC4356506

[B44] Gogtay N. Thompson P. M. (2010). Mapping gray matter development: implications for typical development and vulnerability to psychopathology. Brain Cogn. 72, 6–15 10.1016/j.bandc.2009.08.009 19796863 PMC2815268

[B45] Gogtay N. Vyas N. S. Testa R. Wood S. J. Pantelis C. (2011). Age of onset of schizophrenia: perspectives from structural neuroimaging studies. Schizophr. Bull. 37, 504–513 10.1093/schbul/sbr030 21505117 PMC3080674

[B46] Gracie A. Freeman D. Green S. Garety P. A. Kuipers E. Hardy A. (2007). The association between traumatic experience, paranoia and hallucinations: a test of the predictions of psychological models. Acta Psychiatr. Scand.116, 280–289 10.1111/j.1600-0447.2007.01011.x 17803758

[B47] Green M. F. Hugdahl K. Mitchell S. (1994). Dichotic listening during auditory hallucinations in schizophrenia. Am. J. Psychiatry 151, 357–362 8109643 10.1176/ajp.151.3.357

[B48] Grimby A. (1998). Hallucinations following the loss of a spouse: common and normal events amongthe elderly. J. Clin. Gerontol. 4, 65–74

[B49] Gurvich C. Georgiou-Karistianis N. Fitzgerald P. B. Millist L. White O. B. (2007). Inhibitory control and spatial working memory in Parkinson's disease. Mov. Disord. 22, 1444–1450 10.1002/mds.21510 17516454

[B50] Hackman D. A. Farah M. J. (2009). Socioeconomic status and the developing brain. Trends Cogn. Sci. 13, 65–73 10.1016/j.tics.2008.11.003 19135405 PMC3575682

[B51] Haddock G. McCarron J. Tarrier N. Faragher E. B. (1999). Scales to measure dimensions of hallucinations and delusions: the psychotic symptom rating scales (PSYRATS). Psychol. Med. 29, 879–889 10.1017/S0033291799008661 10473315

[B52] Hammersley P. Dias A. Todd G. Bowen-Jones K. Reilly B. Bentall R. P. (2003). Childhood trauma and hallucinations in bipolar affective disorder: a preliminary investigation. Br. J. Psychiatry 182, 543–547 10.1192/02-151 12777347

[B53] Healey M. K. Campbell K. L. Hasher L. (2008). Cognitive aging and increased distractibility: costs and potential benefits. Prog. Brain Res. 169, 353–363 10.1016/S0079-6123(07)00022-2 18394486

[B54] Hoffman R. E. Varanko M. Gilmore J. Mishara A. L. (2008). Experiential features used by patients with schizophrenia to differentiate ‘voice’ from ordinary verbal thought. Psychol. Med. 38, 1167–1176 10.1017/S0033291707002395 18047771

[B55] Howes O. D. Bose S. K. Turkheimer F. Valli I. Egerton A. Valmaggia L. R. (2011). Dopamine synthesis capacity before onset of psychosis: a prospective [18]-DOPA PET imaging study. Am. J. Psychiatry 168, 1311–1317 10.1176/appi.ajp.2011.11010160 21768612 PMC3682447

[B56] Howes O. D. Kambeitz J. Kim E. Stahl D. Slifstein M. Abi-Dargham A. (2012). The nature of dopamine dysfunction in schizophrenia and what this means for treatment. Meta-analysis of imaging studies. Arch. Gen. Psychiatry 69, 776–786 10.1001/archgenpsychiatry.2012.169 22474070 PMC3730746

[B57] Howes O. D. Kapur S. (2009). The dopamine hypothesis of schizophrenia: version III—the final common pathway. Schizophr. Bull. 35, 549–562 10.1093/schbul/sbp006 19325164 PMC2669582

[B58] Howes O. D. Montgomery A. J. Asselin M. C. Murray R. M. Valli I. Tabraham P. (2009). Elevated striatal dopamine function linked to prodromal signs of schizophrenia. Arch. Gen. Psychiatry 66, 13–20 10.1001/archgenpsychiatry.2008.514 19124684

[B59] Howes O. D. Shotbolt P. Bloomfield M. Daalman K. Demjaha A. Diederen K. M. (2013). Dopaminergic function in the psychosis spectrum: an [18F]-DOPA imaging study in healthy individuals with auditory hallucinations. Schizophr. Bull. 39, 807–814 10.1093/schbul/sbr195 22282457 PMC3686439

[B60] Hugdahl K. Andersson L. (1986). The “forced-attention paradigm” in dichotic listening to CV-syllables: a comparison between adults and children. Cortex 22, 4l7–432 10.1016/S0010-9452(86)80005-3 3769494

[B61] Hugdahl K. Løberg E.-M. Falkenberg L. E. Johnsen E. Kompus K. Kroken R. (2012). Auditory verbal hallucinations in schizophrenia as aberrant lateralized speech perception: evidence from dichotic listening. Schizophr. Res. 140, 59–64 10.1016/j.schres.2012.06.019 22796149

[B62] Hugdahl K. Løberg E.-M. Jørgensen H. Specht K. Steen V. M. Wageningen H. (2008). Auditory hallucinations in schizophrenia: the role of cognitive, brain structural and genetic disturbances in the left temporal lobe. Front. Hum. Neurosci. 1:6 10.3389/neuro.09/006 18958220 PMC2525988

[B63] Hugdahl K. Løberg E. M. Nygård M. (2009b). Left temporal lobe structural and functional abnormality underlying auditory hallucinations in schizophrenia. Front. Neurosci. 3, 34–45 10.3389/neuro.01.001.2009 19753095 PMC2695389

[B64] Hugdahl K. Nygård M. Falkenberg L. E. Kompus K. Westerhausen R. Kroken R. (2013). Failure of attention focus and cognitive control in schizophrenia patients with auditory verbal hallucinations: evidence from dichotic listening. Schizophr. Res. 147, 301–309 10.1016/j.schres.2013.04.005 23664588

[B65] Hugdahl K. Westerhausen R. Alho K. Medvedev S. Laine M. Hämäläinen H. (2009a). Attention and cognitive control: unfolding the dichotic listening story. Scand. J. Psychol. 50, 11–22 10.1111/j.1467-9450.2008.00676.x 18705670

[B66] Insel T. (2013). “Transforming Diagnosis,” The National Institute of Mental Health Director's BlogPosts. Available online at: http://www.nimh.nih.gov/about/director/2013/index.shtml

[B67] Insel T. R. Cuthbert B. Garvey M. Heinssen R. Pine D. S. Quinn K. (2010). Research Domain Criteria (RDoC): towards a new classification framework for research on mental disorders. Am. J. Psychiatry 167, 748–751 10.1176/appi.ajp.2010.09091379 20595427

[B68] Jardri R. Cachia A. Thomas P. Pins D. (2012). The Neuroscience of Hallucinations. New York, NY: Springer Science + Business Media

[B69] Jardri R. Pouchet A. Pins D. Thomas P. (2011). Cortical activations during auditory verbal hallucinations in schizophrenia: a coordinate-based meta-analysis. Am. J. Psychiatry 168, 73–81 10.1176/appi.ajp.2010.09101522 20952459

[B70] Jessop M. Scott J. Nurcombe B. (2008). Hallucinations in adolescent in-patients with post-traumatic stress disorder and schizophrenia: similarities and differences. Australas. Psychiatry 16, 268–272 10.1080/10398560801982580 18608156

[B71] Johnsen E. Hugdahl K. Fusar-Poli P. Kroken R. A. Kompus K. (2013). Neuropsychopharmacology of auditory hallucinations: insights from pharmacological MRI and perspectives for future research. Expert Rev. Neurother. 13, 23–36 10.1586/ern.12.147 23253389

[B72] Jung W. H. Borgwardt S. Fusar-Poli P. Kwon S. J. (2012). Gray matter volumetric abnormalities associated with the onset of psychosis. Front. Psychiatry 3:101 10.3389/fpsyt.2012.00101 23227013 PMC3512053

[B73] Kapur S. Mamo D. (2003). Half a century of antipsychotics and still a central role for dopamine D2 receptors. Prog. Neuropsychopharmol. Biol. Psychiatry 27, 1081–1090 10.1016/j.pnpbp.2003.09.004 14642968

[B74] Kay S. R. Fiszbein A. Opfer L. A. (1987). The Positive and Negative Syndrome Scale (PANSS) for schizophrenia. Schizophr. Bull. 13, 261–276 10.1093/schbul/13.2.261 3616518

[B75] Kaymaz N. Drukker M. Lieb R. Wittchen H. U. Werbeloff N. Weiser M. (2012). Do subthreshold psychotic experiences predict clinical outcomes in unselected non-help-seeking population-based samples? A systematic review and meta-analysis, enriched with new results. Psychol. Med. 42, 2239–2253 10.1017/S0033291711002911 22260930

[B76] Kelleher I. Connor D. Clarke M. C. Devlin N. Harley M. Cannon M. (2012a). Prevalence of psychotic symptoms in childhood and adolescence: a systematic review and meta-analysis of population-based studies. Psychol. Med. 42, 1857–1863 10.1017/S0033291711002960 22225730

[B77] Kelleher I. Corcoran P. Keeley H. Wigman J. T. Devlin N. Ramsay H. (2013). Psychotic symptoms and population risk for suicide attempt: a prospective cohort study. JAMA Psychiatry 70, 940–948 10.1001/jamapsychiatry.2013.140 23863946

[B78] Kelleher I. Keeley H. Corcoran P. Lynch F. Fitzpatrick C. Devlin N. (2012b). Clinico-pathological significance of psychotic symptoms in non-psychotic young people: evidence from 4 population studies. Br. J. Psychiatry 201, 26–32 10.1192/bjp.bp.111.101543 22500011

[B79] Kompus K. Westerhausen R. Hugdahl K. (2011). The “paradoxical” engagement of the primary auditory cortex in patients with auditory verbal hallucinations: a meta-analysis of functional neuroimaging studies. Neuropsychologia 49, 3361–3369 10.1016/j.neuropsychologia.2011.08.010 21872614

[B80] Kringelbach M. I. (2005). The human orbitofrontal cortex: linking reward to hedonic experience. Nat. Rev. Neurosci. 6, 691–702 10.1038/nrn1747 16136173

[B81] Kubota M. Miyata J. Sasamoto A. Sugihara G. Yoshida H. Kawada R. (2012). Thalamocortical disconnection in the orbitofrontal region associated with cortical thinning in schizophrenia. JAMA Psychiatry 70, 12–21 10.1001/archgenpsychiatry.2012.1023 22945538

[B82] Laruelle M. Abi-Dargham A. (1999). Dopamine as the wind of the psychotic fire: new evidence from brain imaging studies. J. Psychopharmacol. 13, 358–371 10.1177/026988119901300405 10667612

[B83] Laurens K. R. Hobbs M. J. Sunderland M. Green M. J. Mould G. L. (2012). Psychotic-like experiences in a community sample of 8000 children aged 9 to 11 years: an item response theory analysis. Psychol. Med. 42, 1495–1506 10.1017/S0033291711002108 21999924

[B85] Linscott R. J. van Os J. (2013). An updated and conservative systematic review and meta-analysis of epidemiological evidence on psychotic experiences in children and adults: on the pathway from proneness to persistence to dimensional expression across mental disorders. Psychol. Med. 43, 1133–1149 10.1017/S0033291712001626 22850401

[B86] Løberg E. M. Hugdahl K. Green M. F. (1999). Hemispheric asymmetry in schizophrenia: a “dual deficits” model. Biol. Psychiatry 45, 76–81 10.1016/S0006-3223(98)00219-4 9894578

[B87] Longden E. (2010). Making sense of voices: a personal story of recovery. Psychosis 2, 255–259 10.1080/17522439.2010.512667

[B88] Luna B. Padmanabhan A. O'Hearn K. (2010). What has fMRI told us about the development of cognitive control through adolescence? Brain Cogn. 72, 101–113 10.1016/j.bandc.2009.08.005 19765880 PMC2815087

[B89] Luna B. Paulsen D. J. Padmanabhan A. Geier C. (2013). The teenage brain: cognitive control and motivation. Curr. Dir. Psychol. Sci. 22, 94–100 10.1177/0963721413478416 PMC4285389 25574074

[B90] Martinez D. Orlowska D. Narendran R. Slifstein M. Liu F. Kumar D. (2010). Dopamine type 2/3 receptor availability in the striatum and social status in human volunteers. Biol. Psychiatry 67, 275–278 10.1016/j.biopsych.2009.07.037 19811777 PMC2812584

[B91] Matthys W. Vanderschuren L. J. Schutter D. J. (2013). The neurobiology of oppositional defiant disorder and conduct disorder: altered functioning in three mental domains. Dev. Psychopathol. 25, 193–207 10.1017/S0954579412000272 22800761

[B92] McCarthy-Jones S. (2013). Hearing Voices. The Histories, Causes and Meanings of Auditory Verbal Hallucinations. Cambridge, MA: Cambridge University Press

[B93] Meads D. M. Bentall R. P. (2008). Rasch analysis and item reduction of the hypomanic personality scale. Pers. Indiv. Differ. 44, 1772–1783 10.1016/j.paid.2008.02.009

[B94] Miller E. K. Cohen J. D. (2001). An integrative theory of prefrontal cortex function. Annu. Rev. Neurosci. 24, 167–202 10.1146/annurev.neuro.24.1.167 11283309

[B95] Moran M. E. Hulshoff Pol H. Gogtay N. (2013). A family affair: brain abnormalities in siblings of patients with schizophrenia. Brain 136, 3215–3226 10.1093/brain/awt116 23698280 PMC3808683

[B96] Mullins P. G. McGonigle D. J. O'Gorman R. L. Puts N. A. Vidyasagar R. Evans C. J. (2014). Current practice in the use of MEGA-PRESS spectroscopy for the detection of GABA. Neuroimage 86, 43–52 10.1016/j.neuroimage.2012.12.004 23246994 PMC3825742

[B97] National Institute of Mental Health. (2013). “The National Institute of Mental Health Strategic Plan.” Available online at: http://www.nimh.nih.gov/research-funding/rdoc/nimh-research-domain-criteria-rdoc.shtml

[B98] Nestor P. G. Nakamura M. Niznikiewicz M. Thompson E. Levitt J. J. Choate V. (2013). In search of the functional neuroanatomy of sociality: MRI subdivisions of orbital frontal cortex and social cognition. Soc. Cogn. Affect. Neurosci. 8, 460–467 10.1093/scan/nss018 22345366 PMC3624957

[B99] Niendam T. A. Laird A. R. Ray K. L. Dean Y. M. Glahn D. C. Carter C. S. (2012). Meta-analytic evidence for a superordinate cognitive control network subserving diverse executive function. Cogn. Affect. Behav. Neurosci. 12, 241–268 10.3758/s13415-011-0083-5 22282036 PMC3660731

[B100] Paulik G. Badcock J. C. Maybery M. (2007). Poor intentional inhibition in individuals predisposed to hallucinations. Cogn. Neuropsychiatry 12, 457–470 10.1080/13546800701394329 17691002

[B101] Paulik G. Badcock J. C. Maybery M. (2008). Dissociating the components of inhibitory control involved in predisposition to hallucinations. Cogn. Neuropsychiatry 13, 33–46 10.1080./13546800701775683 18092224

[B102] Pihan H. Gutbrod K. Baas U. Schnider A. (2004). Dopamine inhibition and the adaptation of behaviour to ongoing reality. Neuroreport 15, 709–712 10.1097/00001756-200403220-00027 15094481

[B103] Puts N. J. A. Edden R. A. E. (2012). *In vivo* magnetic resonance spectroscopy of GABA: a methodological review. Prog. Nucl. Magn. Reson. Spectrosc. 60, 29–41 10.1016/j.pnmrs.2011.06.001 22293397 PMC3383792

[B104] Rossler W. Hengartner R. P. Ajdacic-Gross V. Haker H. Gamma A. Angst J. (2011). Sub-clinical psychosis symptoms in young adults are risk factors for subsequent common mental disorders. Schizophr. Res. 131, 18–23 10.1016/j.schres.2011.06.019 21757323

[B105] Rossler W. Riecher-Rossler A. Angst J. Murray R. Gamma A. Eich D. (2007). Psychotic experiences in the general population: a twenty-year prospective community study. Schizophr. Res. 92, 1–14 10.1016/j.schres.2007.01.002 17363221

[B106] Sanjuan J. Molto M. D. Tolosa A. (2013). Candidate genes in the expression of psychotic symptoms: a focus on hallucinations, in The Neuroscience of Hallucinations, eds Jardri R. Cachia A. Thomas P. Pins D. (New York, NY: Springer), 231–252

[B107] Scarmeas N. Brandt J. Albert M. Hadjigeorgiou G. Papadimitriou A. Dubois B. (2005). Delusions and hallucinations are associated with worse outcome in Alzheimer disease. Arch. Neurol. 62, 601–1608 10.1001/archneur.62.10.1601 16216946 PMC3028538

[B108] Schnider A. (2003). Spontaneous confabulations and the adaptation of thought to ongoing reality. Nat. Rev. Neurosci. 4, 662–671 10.1038/nrn1179 12894241

[B109] Schnider A. (2008). The Confabulating Mind. How the brain creates reality. Oxford: Oxford University Press 10.1093/med/9780199206759.001.0001

[B110] Schnider A. (2013). Orbitofrontal reality filtering. Front. Behav. Neurosci.7:67 10.3389/fnbeh.2013.00067 23772208 PMC3677127

[B111] Schnider A. Guggisberg A. Nahum D. Gabriel D. Morand S. (2010). Dopaminergic modulation of rapid reality adaptation in thinking. Neuroscience 167, 583–587 10.1016/j.neuroscience.2010.02.044 20219638

[B112] Schnider A. Treyer V. Buck A. (2000). Selection of currently relevant memories by the human posterior medial orbitofrontal cortex. J. Neurosci. 20, 5880–5884 10908632 10.1523/JNEUROSCI.20-15-05880.2000 PMC6772539

[B113] Schroeder K. Fisher H. L. Schafer I. (2013). Psychotic symptoms in patients with borderline personality disorder: prevalence and clinical management. Curr. Opin. Psychiatry 26, 113–119 10.1097/YCO.0b013e32835a2ae7 23168909

[B114] Shinn A. K. Pfaff D. Young S. Lewandowski A. K. Cohen B. M. Ongür D. (2012). Auditory hallucinations in a cross-diagnostic sample of psychotic disorder patients: a descriptive, cross-sectional study. Compr. Psychiatry 53, 718–726 10.1016/j.comppsych.2011.11.003 22197213 PMC3314103

[B115] Silbersweig D. A. Stern E. Frith C. Cahill C. Holmes A. Grootonk S. (1995). A functional neuroanatomy study of hallucinations in schizophrenia. Nature 378, 176–179 10.1038/378176a0 7477318

[B116] Slotema C. W. Daalman K. Blom J. D. Diederen K. M. Hoek H. W. Sommer I. E. (2012). Auditory verbal hallucinations in patients with borderline personality disorder are similar to those in schizophrenia. Psychol. Med. 42, 1873–1878 10.1017/S0033291712000165 22336487

[B117] Soveri A. Tallus J. Laine M. Nyberg L. Bäckman L. Hugdahl K. (2013). Modulation of auditory attention by training: evidence from dichotic listening. Exp. Psychol. 60, 44–52 10.1027/1618-3169/a000172 22935330

[B118] Stephane M. (2013). Auditory verbal hallucinations result from combinatoric associations of multiple neural events. Front. Hum.Neurosci. 7:239 10.3389/fnhum.2013.00239 23755004 PMC3668292

[B119] Stokes P. A. Shotbolt P. Mehta M. A. Turkheimer E. Benecke A. Copeland C. (2013). Nature or nurture? Determining the heritability of Human striatal dopamine function: an [18F]-DOPA PET study. Neuropsychopharmacology 38, 485–491 10.1038/npp.2012.207 23093224 PMC3547199

[B120] Stroop J. R. (1935). Studies of interference in serial verbal reactions. J. Exp. Psychol. 18, 643–662 10.1037/h0054651

[B121] Sun J. Jayathilake K. Zhao Z. Meltzer H. (2012). Investigating association of four gene regions (GABRB3, MAOB, PAH, and SLC6A4) with five symptoms in schizophrenia. Psychiatry Res. 198, 202–206 10.1016/j.psychres.2011.12.035 22414661

[B122] Thomsen T. Specht K. Rimol L. M. Hammar Å. Nyttingnes J. Ersland L. (2004). Brain localization of attentional control in different age groups by combining functional and structural MRI. Neuroimage 22, 912–919 10.1016/j.neuroimage.2004.02.015 15193622

[B123] Turvey C. L. Schultz S. K. Arndt S. Ellingrod V. Wallace R. Herzog R. (2001). Caregiver report of hallucinations and paranoid delusions in elders aged 70 or older. Int. Psychogeriatr. 13, 241–249 10.1017/S1041610201007621 11495398

[B124] van Rossum I. Dominguez M. D. Lieb R. Wittchen H. U. van Os J. (2011). Affective dysregulation and reality distortion: a 10-year prospective study of their association and clinical relevance. Schizophr. Bull. 37, 561–571 10.1093/schbul/sbp101 19793794 PMC3080695

[B125] Varese F. Barkus E. Bentall R. P. (2012). Dissociation mediates the relationship between childhood trauma and hallucination-proneness. Psychol. Med. 42, 1025–1103 10.1017/S0033291711001826 21896238

[B126] Varghese D. Scott J. Welham J. Bor W. Najman J. O'Callaghan M. (2011). Psychotic-like experiences in major depression and anxiety disorders: a population-based survey in young adults. Schizophr. Bull. 37, 389–393 10.1093/schbul/sbp083 19687152 PMC3044630

[B127] Waters F. Allen P. Aleman A. Fernyhough C. Woodward T. S. Badcock J. C. (2012). Auditory hallucinations in schizophrenia and non-schizophrenia populations: a review and integrated model of cognitive mechanisms. Schizophr. Bull. 38, 683–693 10.1093/schbul/sbs045 22446568 PMC3406530

[B128] Waters F. Badcock J. C. Maybery M. Michie P. (2003). Inhibition in schizophrenia: association with auditory hallucinations. Schizophr. Res. 62, 275–280 10.1016/S0920-9964(02)00358-4 12837525

[B129] Whelan R. Conrod P. J. Poline J.-P. Lourdusamy A. Banaschewski T. Barker G. J. (2012). Adolescent impulsivity phenotypes characterized by distinct brain networks. Nat. Neurosci. 15, 920–925 10.1038/nn.3092 22544311

[B130] Wigman J. T. van Nierop M. Vollebergh W. A. Lieb R. Beesdo-Baum K. Wittchen H. U. (2012). Evidence that psychotic symptoms are prevalent in disorders of anxiety and depression, impacting on illness onset, risk, and severity: implications for diagnosis and ultra-high risk research. Schizophr. Bull. 38, 247–257 10.1093/schbul/sbr196 22258882 PMC3283146

[B131] Wilson-Mendenhall C. D. Feldman Barrett L. Barsalou L. W. (2013). Neural evidence that human emotions share core affective properties. Psychol. Sci. 24, 947–956 10.1177/0956797612464242 23603916 PMC4015729

[B132] Woodruff P. Wright I. Bullmore E. Brammer M. Howard R. Williams S. C. (1997). Auditory hallucinations and the temporal cortical response to speech in schizophrenia: a functional magnetic resonance imaging study. Am. J. Psychiatry 154, 1676–1682 9396945 10.1176/ajp.154.12.1676

